# Identifying genes that mediate anthracyline toxicity in immune cells

**DOI:** 10.3389/fphar.2015.00062

**Published:** 2015-04-15

**Authors:** Amber Frick, Oscar T. Suzuki, Cristina Benton, Bethany Parks, Yuri Fedoriw, Kristy L. Richards, Russell S. Thomas, Tim Wiltshire

**Affiliations:** ^1^Division of Pharmacotherapy and Experimental Therapeutics, Eshelman School of Pharmacy, University of North CarolinaChapel Hill, NC, USA; ^2^The Hamner Institutes for Health Sciences, Research Triangle ParkNC, USA; ^3^Department of Pathology and Laboratory Medicine, School of Medicine, University of North CarolinaChapel Hill, NC, USA; ^4^Lineberger Comprehensive Cancer Center, School of Medicine, University of North CarolinaChapel Hill, NC, USA; ^5^Department of Genetics, School of Medicine, University of North CarolinaChapel Hill, NC, USA; ^6^National Center for Computational Toxicology, U.S. Environmental Protection Agency, Research Triangle ParkNC, USA

**Keywords:** pharmacogenomics, genome-wide association studies, candidate genes, amyloid precursor protein, anthracyclines, immune cells

## Abstract

The role of the immune system in response to chemotherapeutic agents remains elusive. The interpatient variability observed in immune and chemotherapeutic cytotoxic responses is likely, at least in part, due to complex genetic differences. Through the use of a panel of genetically diverse mouse inbred strains, we developed a drug screening platform aimed at identifying genes underlying these chemotherapeutic cytotoxic effects on immune cells. Using genome-wide association studies (GWAS), we identified four genome-wide significant quantitative trait loci (QTL) that contributed to the sensitivity of doxorubicin and idarubicin in immune cells. Of particular interest, a locus on chromosome 16 was significantly associated with cell viability following idarubicin administration (*p* = 5.01 × 10^−8^). Within this QTL lies *App*, which encodes amyloid beta precursor protein. Comparison of dose-response curves verified that T-cells in *App* knockout mice were more sensitive to idarubicin than those of C57BL/6J control mice (*p* < 0.05). In conclusion, the cellular screening approach coupled with GWAS led to the identification and subsequent validation of a gene involved in T-cell viability after idarubicin treatment. Previous studies have suggested a role for *App* in *in vitro* and *in vivo* cytotoxicity to anticancer agents; the overexpression of *App* enhances resistance, while the knockdown of this gene is deleterious to cell viability. Further investigations should include performing mechanistic studies, validating additional genes from the GWAS, including *Ppfia1* and *Ppfibp1*, and ultimately translating the findings to *in vivo* and human studies.

## Introduction

The role of the immune system in cancer development is well established with the evasion of immune elimination described as one of Hanahan and Weinberg's hallmarks of cancer development (Hanahan and Weinberg, 2011). Developing tumors commonly avoid immune surveillance by inducing an immunosuppressive tumor microenvironment with regulatory T-cells, myeloid-derived suppressor cells, alternatively activated macrophages, and tolerant dendritic cells (Alizadeh and Larmonier, 2014). Thus, the induction, potency, and persistence of the patient's functional immune system is critical to combating tumor advancement (Raval et al., 2014).

The generation of an efficacious clinical antitumor response depends upon the successful initiation of several immune processes. In this regard, the adaptive immune system has been described as an ideal anticancer agent with features including diversity, specificity, and memory. Recent advances in immune-based therapeutic approaches have focused on boosting the adaptive antitumor immune response using various approaches, including vaccination, adoptive T-cell therapy, anti-tumor antibodies, and the advent of immune checkpoint blockade agents (Hodi et al., 2010; Brahmer et al., 2012; Kantoff et al., 2012; Topalian et al., 2012; Kalos and June, 2013). Clinically, monitoring T- and B-cell response may prove useful in correlating specific immune responses to patient outcomes (Raval et al., 2014). For instance, patients with denser T-cell infiltrates in a variety of cancer tumors have better clinical responses to traditional, cytotoxic chemotherapy agents compared to patients with smaller infiltrates (Galluzzi et al., 2012). Some cytotoxic chemotherapeutics, such as anthracyclines, promote immunogenic cell death by releasing molecules such as calreticulin, which subsequently primes T-cells to elicit an antitumor Th1 phenotype (Ma et al., 2011; Mattarollo et al., 2011). Therefore, assessing the functionality of the immune system is crucial for evaluating clinical responses to cytotoxic chemotherapy (Vanneman and Dranoff, 2012).

Previous studies have noted intersubject variability in chemotherapy-induced cytotoxicity within the immune system (van Kuilenburg et al., 2000; Stocco et al., 2008; Ross et al., 2011). Although several genes have been linked to the toxicity of anticancer chemotherapy on the innate immune system (i.e., neutropenia), the role of pharmacogenomics in the cytotoxicity of the adaptive immune system requires further investigation. Here, we examined the underlying genetic components that may be responsible for the differential immune cell sensitivity to anticancer drugs. A model organism approach was used to evaluate pharmacotherapeutic response, as the effects of chemotherapy on the normal immune system are difficult to ascertain in humans. We previously developed a cell-based screen using immune cells from 36 inbred mouse strains to measure phenotypic differences in immune cell sensitivity to anticancer therapeutics (Frick et al., 2015). We were able to identify robust interstrain variation in T- and B-cell viability to cytotoxic anthracycline agents, doxorubicin and idarubicin. This *in vitro* pharmacogenomics screen was also developed to facilitate identification of genetic biomarkers involved in immune cytotoxicity pathways. Thus, the aims of this study were to identify quantitative trait loci (QTL) that contribute to the sensitivity of T- and B-cells to the described anthracyclines using genome-wide association studies (GWAS) and to prioritize and validate candidate genes. Following GWAS, we identified a candidate gene, *App* (encoding amyloid beta precursor protein) that was further shown to be involved in mediating T-cell sensitivity to idarubicin.

## Materials and methods

### Phenotype determination

The methods and results of our drug-screening platform in normal, non-cancerous, murine immune cells have been previously described (Frick et al., 2015). Briefly, splenocytes were collected from a panel of 36 inbred mouse strains (*n* = 4 per strain, 129S1/SvImJ, 129X1/SvJ, A/J, AKR/J, BALB/cByJ, BTBR *T*^+^ *Itpr3^tf^*/*J*, BUB/BnJ, C3H/HeJ, C57BLKS/J, C57BL/6J, C57BR/cdJ, C58/J, CBA/J, CZECHII/EiJ, DBA/2J, FVB/NJ, I/LnJ, KK/HiJ, LG/J, LP/J, MA/MyJ, NOD/LtJ, NON/LtJ, NZB/BINJ, NZO/HiLtJ, NZW/LacJ, PERA/EiJ, PL/J, PWD/PhJ, PWK/PhJ, RIIIS/J, SEA/GnJ, SJL/J, SM/J, SWR/J, and WSB/EiJ), aged 10–12 weeks, obtained from The Jackson Laboratory Mouse Diversity Panel (Bar Harbor, ME). Splenocytes were isolated using standard procedures; spleens were excised and mechanically dissociated into a single-cell suspension, after which red blood cells were removed using ammonium-chloride-potassium lysing buffer (Gibco, Grand Island, NY, USA). Splenocytes at a density of 100,000 cells per mL and volume of 100 μL per well were then exposed to nine half-logarithmic concentrations of doxorubicin, idarubicin (Sigma-Aldrich, Milwaukee, WI, USA), BEZ-235 (provided by Novartis, Inc.), and selumetinib (ChemieTek, Indianapolis, IN, USA) ranging from 0.01 to 100 μM. At 4 h post-treatment, cells were sequentially incubated with physiological indicator dyes [i.e., 3.75 μL (0.19 μg) 7-AAD (BD Biosciences, San Jose, CA, USA), 3.75 μM CellEvent™ Caspase-3/7 Green Detection Reagent, and 125 nM Mitotracker® Deep Red (Invitrogen, Carlsbad, CA, USA) per 100 μL well] for 30 min at 37°C and 5% CO_2_ and cell surface marker antibodies [i.e., 0.05 μg V500 Syrian hamster anti-mouse CD-3e, 0.1 μg APC-H7 rat anti-mouse CD-19, 0.1 μg V450 rat anti-mouse CD-11b, and 0.1 μg PE-Cy7 rat anti- mouse Ly-6G per 100 μL well (all antibodies were obtained from BD Biosciences)] for 30 min at 4°C and then fixed with 4% paraformaldehyde (Thermo Fisher Scientific, Pittsburgh, PA, USA) for 15 min at room temperature. Samples were analyzed by flow cytometry using a BD FACSCanto™ II flow cytometer (BD Biosciences) equipped with three lasers (405 nm, 488 nm, and 640 nm) and Flow Jo software version X (TreeStar, Ashland, OR, USA). Dose-response curves with response normalized to the zero dose as a function of log concentration were generated. After detecting immune cell populations of interest (e.g., CD19+ B-cells, CD3e+ T-cells, and CD11b+ monocytes), cells positive for physiological indicator dyes in each subpopulation were gated. Dose-response curves with response normalized to the zero dose as a function of log concentration were generated using GraphPad Prism 6 (La Jolla, CA) and the Hill equation:

$$f(x)=Max-\frac{Max-Min}{1+{\left(\frac{x}{IC50}\right)}^{\gamma }}$$

where *Max* is the maximum asymptote, *Min* is the minimum asymptote, γ is the Hill slope, and *x* is the drug concentration (Beam and Motsinger-Reif, 2013). Heritability or the percent of variability likely due to genetics was calculated by comparing intra- and interstrain variation in percent viability. The proportion of phenotype variation attributable to genetics was estimated with broad-sense heritability. Intrastrain correlations were estimated by

$$r1=\frac{MSB-MSW}{MSB+\left(n+1\right)MSW}$$

where *r*1 is the intrastrain correlation estimate, *MSB* is the mean square of the between-strain comparison, *MSW* is the mean square of the within-strain correlation, and n is the number of animals per strain (Nichols et al., 2014). Viability measurements of B-cells and T-cells exposed to the anthracyclines provided the most heritable phenotypes in this screen and thus underwent further QTL mapping analysis (Frick et al., 2015).

### Quantitative trait loci (QTL) mapping

GWAS were performed for half maximal inhibitory concentration (IC_50_) values and individual drug concentrations that corresponded to cell viability for splenic B-cells and T-cells exposed to doxorubicin and idarubicin. SNPster and efficient mixed-model association (EMMA) algorithms, which are well described elsewhere, were used for QTL mapping (McClurg et al., 2007; Kang et al., 2008). Briefly, SNPster performs QTL mapping analysis from an inferred haplotype structure determined by overlapping 3-SNP windows for each strain. Using One-Way ANOVA, an *F*-statistic is calculated following association analyses of phenotypic values with haplotypes. *p*-values are then estimated by bootstrapping phenotypic values 1 × 10^6^ times, providing a maximum –log(*p*) score of 6.0 (McClurg et al., 2007). EMMA uses *F*-tests for single marker association mapping while accounting for population structure and genetic relatedness (Kang et al., 2008). SNP genotypes used for GWAS were obtained from the Mouse Diversity Array set at the CGDSNPdb website (http://cgd.jax.org/cgsnpdb/) (Yang et al., 2011). The SNP panel was trimmed for redundancy (SNPs showing identical haplotype pattern at a locus), missingness (genotyping call rates <95%), and non-informative nature (SNPs without variation amongst the 36 strains), leaving a panel of 356,596 SNPs. Manhattan plots were visualized using R version 3.1.0 and the UCSC Mouse Genome Browser on the Mouse July 2007 (NCBI37/mm9) Assembly (https://genome.ucsc.edu) (Waterston et al., 2002). The threshold of significance for QTL mapping was adjusted using a conservative Bonferroni correction. QTL were considered significant when the −log(*p*) score was ≥6.85.

### Candidate gene selection

QTL regions that overlapped using both association mapping algorithms, although both were not required to be genome-wide significant [−log(*p*) ≥ 6.85], were further selected for candidate gene selection. Candidate genes were prioritized based on the following: literature evidence for biological involvement with the immune system or anthracycline response, gene expression in spleens and immune cells across strains, correlation between phenotypic values and gene expression levels in spleens and immune cells, similarity in the haplotype structure between the QTL and the candidate gene, presence of potentially deleterious non-synonymous coding SNPs, and apoptotic or immune cell pathway involvement (Supplementary Figure 1) (Moreau and Tranchevent, 2012). Candidate genes were only included if they were expressed in the spleen, the tissue originally assayed to produce our phenotypes of interest. Expression levels were measured in spleens and immune cells from inbred strains of mice using the Affymetrix Mouse Genome 2.0 Array (Santa Clara, CA). Genes were considered expressed if their expression level was greater than 50 for at least one of the strains following data processing with the gcRMA algorithm. Non-synonymous coding SNPs were obtained from dbSNP (http://www.ncbi.nlm.nih.gov/projects/SNP/). The likely effect of amino acid substitutions in protein sequences was determined using PROVEAN (Protein Variation Effect Analyzer) version 1.1.3 (Choi et al., 2012) and PANTHER (Protein Analysis Through Evolutionary Relationships) version 9.0 software (Thomas et al., 2003; Mi et al., 2005). Using PROVEAN, a score of ≤−2.5 indicates a functional effect on the protein. For the PANTHER algorithm, a subSPEC (substitution position-specific evolutionary conservation) score of −3 corresponds to a 50% probability that a score is deleterious (Pdeleterious = 0.5). Chilibot (Chen and Sharp, 2004) (http://www.chilibot.net) was used to search the PubMed literature database for biological relevance of genes with regards to immune cell function or anthracycline response. Ingenuity® Pathway Analysis was used to gage the involvement of genes in apoptotic or various immune function pathways (http://www.ingenuity.com/). The haplotype structure for the interval and for specific genes was reviewed with the Mouse Phylogeny Viewer (https://msub.csbio.unc.edu/) (Wang et al., 2012).

### *App* gene validation

Based on the criteria for candidate gene validation as described above, *App* was chosen for downstream validation studies. An *in vitro* knockout approach was used for validation of App. App knockout (B6.129S7-Apptm1Dbo/J, stock number: 004133) and C57BL/6J control (stock number: 000664) male mice aged 10–12 weeks were obtained from the Jackson Laboratory (Bar Harbor, ME). Other than a reduced body weight of 15–20% less than wildtype age-matched controls, mice homozygous for the targeted allele are viable without blatant physical and behavioral abnormalities at birth. At 14 weeks of age, an age beyond the range we included in our assay, the mice exhibit evidence of reactive gliosis with significantly reduced forearm grip strength and reduced locomotion (Zheng et al., 1995). For the validation study, mice were housed three per cage in polycarbonate cages on a 12 h light/dark cycle (lights on at 0700 h) with access to food and water *ad libitum*. Following 1 week of habituation, splenic immune cells from these knockout and control mice were obtained and underwent the cellular screening as described (Frick et al., 2015). All procedures were approved by the UNC Institutional Animal Care and Use Committee and followed the guidelines set forth by the National Institutes of Health Guide for the Care and Use of Laboratory Animals.

### Statistical analyses

Additional statistical analyses, including Pearson correlations, *t*-tests, and partial *F*-tests, were performed with SAS version 5.2 (Cary, NC) and GraphPad Prism 6 with *p* < 0.05 considered to be statistically significant. Pearson correlations were performed to determine the relatedness between metrics (Brown et al., 2011). Dose-response curves from knockout and control populations were compared using *t*-tests for IC_50_ and viability measurements. Finally, a partial *F*-test was used to determine if there was evidence that the knockout and control data sets differed from each other, necessitating the use of two separate dose-response curves to represent the conglomerate data.

## Results

**Figure 1:**
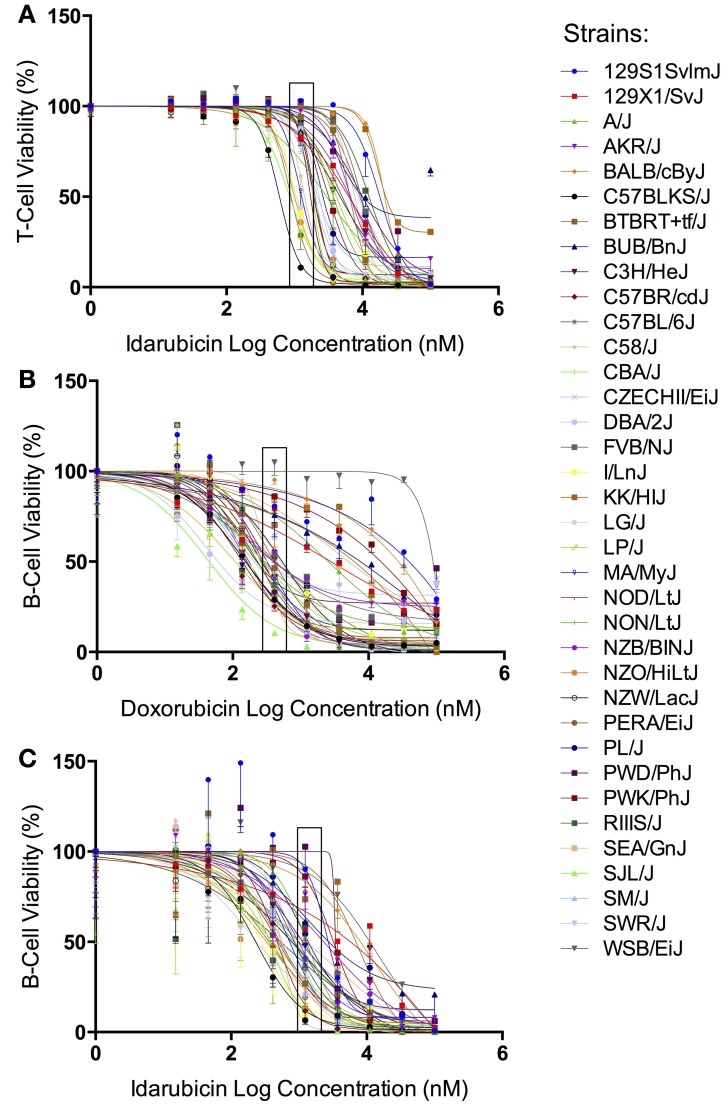
**Phenotypes for GWAS**. Dose-response curves reflecting interstrain variation in viability are shown for T-cells exposed to idarubicin **(A)**, B-cells exposed to doxorubicin **(B)**, and B-cells exposed to idarubicin **(C)**. Thirty-six strains are represented: 129S1/SvImJ, 129X1/SvJ, A/J, AKR/J, BALB/cByJ, BTBR T+ Itpr3tf/J, BUB/BnJ, C3H/HeJ, C57BLKS/J, C57BL/6J, C57BR/cdJ, C58/J, CBA/J, CZECHII/EiJ, DBA/2J, FVB/NJ, I/LnJ, KK/HiJ, LG/J, LP/J, MA/MyJ, NOD/LtJ, NON/LtJ, NZB/BINJ, NZO/HiLtJ, NZW/LacJ, PERA/EiJ, PL/J, PWD/PhJ, PWK/PhJ, RIIIS/J, SEA/GnJ, SJL/J, SM/J, SWR/J, and WSB/EiJ. Concentrations used to generate genome-wide significant QTL (respectively 1, 0.3, and 3 μM) are enclosed with a black box.

The results from our initial *ex vivo* assessment of drug sensitivity phenotypes have been previously discussed (Frick et al., 2015). Although we measured multiple cell health parameters, the cell viability phenotype was most robust and heritable and was subsequently used for GWAS. Figure 1 displays the interstrain phenotypic variation for the most heritable viability phenotypes: T-cells exposed to idarubicin (Figure 1A), B-cells exposed to doxorubicin (Figure 1B), and B-cells exposed to idarubicin (Figure 1C). Several strains repeatedly appeared to be more sensitive (e.g., C57BLKS/J and DBA/2J) or less sensitive (e.g., BALB/cByJ, KK/HiJ, and WSB/EiJ) to the cytotoxic effects of the different anthracycline agents across cell types. The concentrations (respectively 0.3, 1, and 3 μM) contained in black boxes corresponded to the phenotypic values that generated genome-wide significant QTL with respective heritability measurements of 70.6, 87.5, and 85%. The viability measurements at these concentrations significantly correlated with IC_50_ values obtained from our assay with respective Pearson correlations of 0.85942 (*p* < 0.0001), 0.82489 (*p* < 0.0001), and 0.92028 (*p* < 0.0001).

**Figure 2:**
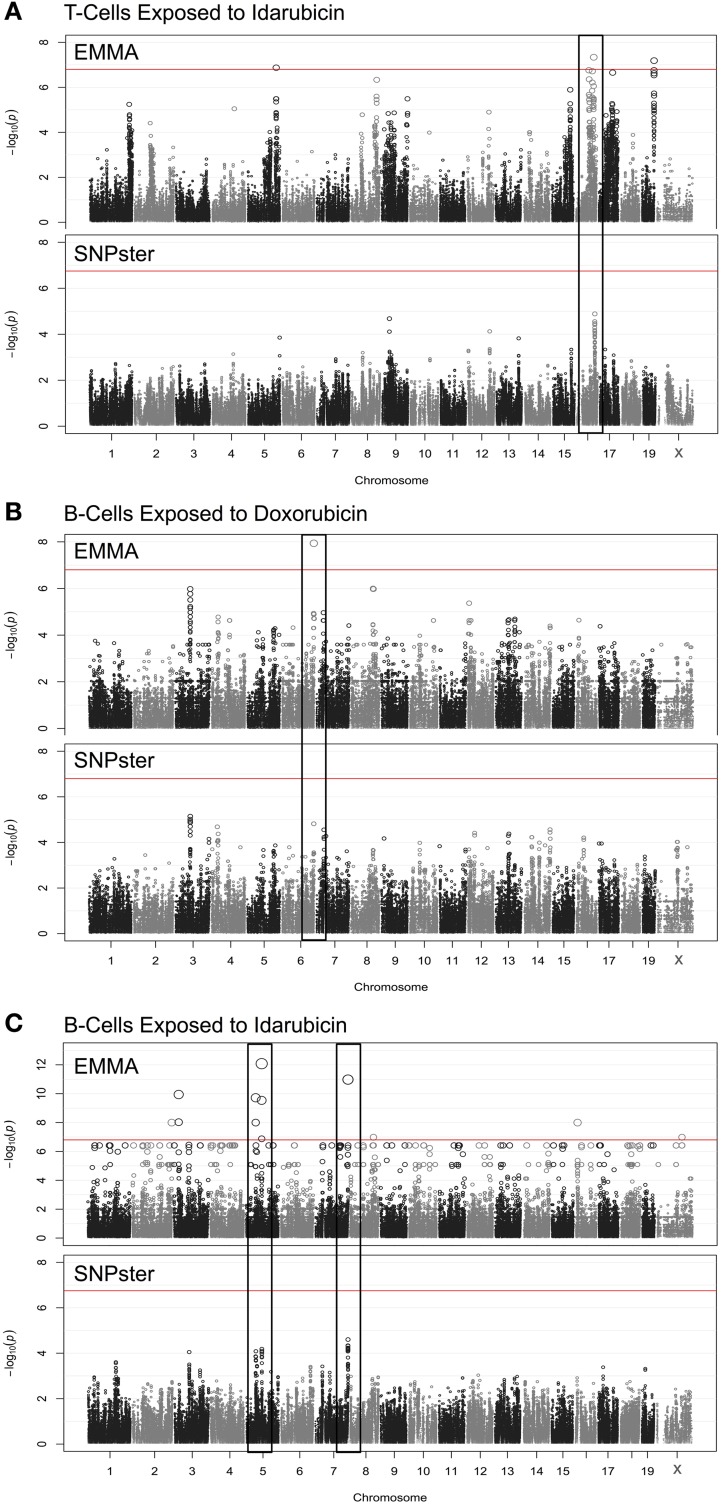
**Manhattan plots for immune cell cytotoxicity to anthracycline agents**. Manhattan plots were obtained from GWAS using EMMA and SNPster algorithms for T-cells exposed to idarubicin **(A)**, B-cells exposed to doxorubicin **(B)**, and B-cells exposed to idarubicin **(C)**. Manhattan plots derived from EMMA are displayed above Manhattan plots obtained from SNPster. The threshold of genome-wide significance (−log(*p*) ≥ 6.85 following Bonferroni correction) is represented by the horizontal red line. The black boxes contain matching QTL peaks obtained from both EMMA and SNPster algorithms respectively on chromosomes 16 **(A)**, 6 **(B)** 5, and 7 **(C)**. The −log(*p*) scores for the respective QTL are 7.34, 7.94, 12.08, and 10.98.

Using a GWA approach, we identified four genome-wide significant QTLs that overlapped using both SNPster and EMMA algorithms: chr16 84.7–85.6 Mb for T-cells exposed to idarubicin (Figure 2A), chr6 146.5–147.5 Mb for B-cells exposed to doxorubicin (Figure 2B), and chr5 74.5–74.9 Mb and chr7 151.4–152.0 Mb for B-cells exposed to idarubicin (Figure 2C) with −log(*p*) scores of 7.34, 7.94, 12.08, and 10.98, respectively. Within these four QTL peaks, there were 25 genes that were further prioritized using the criteria described previously (Supplementary Figure 1). Only 16 out of 25 genes within genome-wide significant peaks were expressed in the spleen (expression level > 50) and were included for prioritization (Supplementary Table 1).

**Figure 3:**
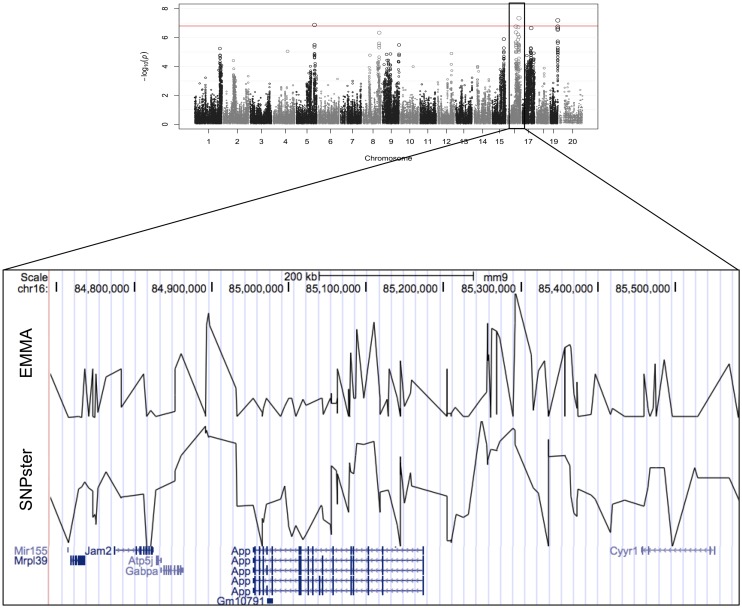
**Genomic region associated with T-cell toxicity following idarubicin exposure**. Potential candidate genes from the Reference Sequence database on chromosome 16 are displayed using Manhattan plots that were generated from both EMMA and SNPster algorithms. The candidate QTL within a 0.9 Mb region is visualized with the UCSC Genome Browser (http://genome.ucsc.edu) with the QTL region derived from EMMA displayed above the QTL region obtained from SNPster.

The viability of splenic T-cells following idarubicin exposure is a robust phenotype exhibiting strong interstrain variability. This phenotype was strongly associated with a 0.9 Mb region (84.7–85.6 Mb) on chromosome 16 containing eight genes, six of which met criteria for candidate gene prioritization (Figure 3). Supplementary Table 1 lists characteristics for all candidate genes across various dose-response phenotypes. Briefly, of the six genes, *App* was one of four (i.e., Atp5j, Gabpa, and Mir155) that is involved in apoptosis and immune cell pathways according to Ingenuity® pathway analysis. Using Chilibot, *App* is the only gene with a known relation to anthracyclines and one of two genes (i.e., *Mir155*) that is associated in the literature with the immune system. The haplotype structure of *App*, *Gabpa*, and *Mrpl39* contained groupings of strains corresponding to sensitive and resistant phenotypes. *App* and *Mrpl39* have potentially deleterious SNPs. *App* is also the only gene under this peak that is differentially expressed (≥2 fold difference in expression levels) across our selected inbred mouse strains in the spleen, CD4+ cells, CD4+ Th1 cells, and macrophages. For these reasons, *App*, encoding amyloid beta precursor protein, was chosen for validation.

**Figure 4:**
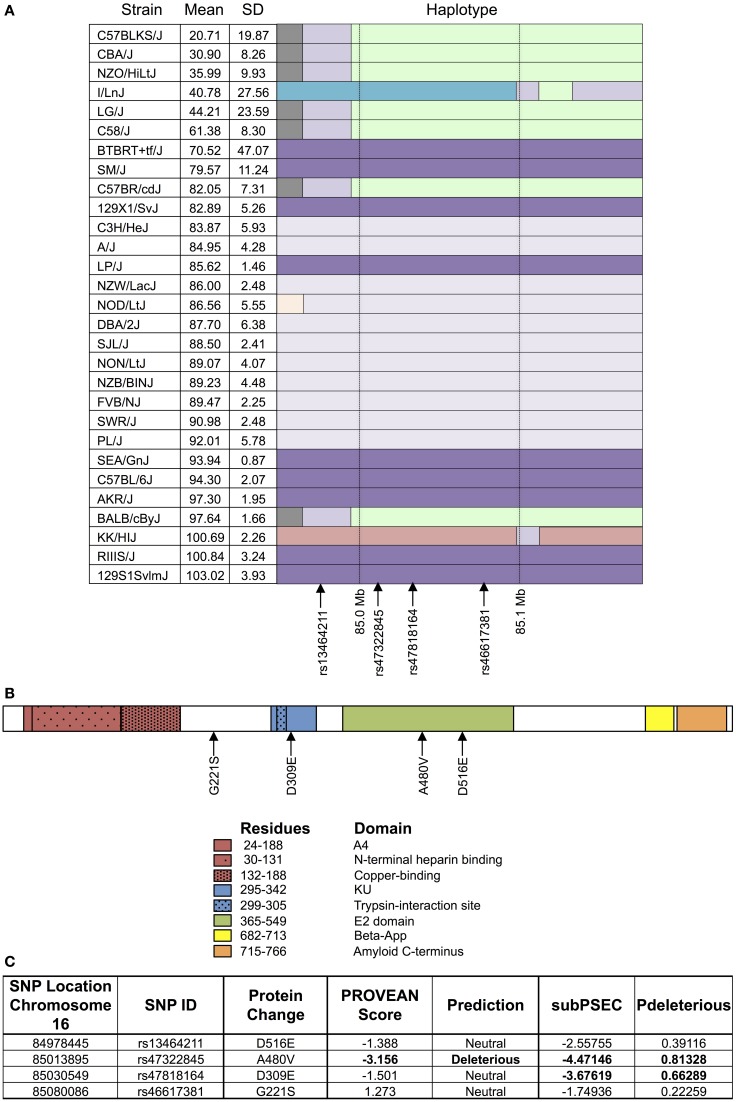
**Haplotype and protein structure of App**. The haplotype structure of the inbred mouse strains within *App* **(A)**, the structure of App **(B)**, and the likelihood of deleterious effects within App due to non-synonymous coding SNPs **(C)** are shown. Strains are arranged in descending order of phenotype (i.e., T-cell viability following exposure to 1 μM idarubicin) from most to least sensitive along with mean, standard deviation, and the haplotype structure (chr16 84.95 Mb–85.17 Mb). The haplotype structure was visualized with the Mouse Phylogeny Viewer (https://msub.csbio.unc.edu/). Within *App*, non-synonymous coding SNPs are indicated by arrows. The structure of *App* is provided with key domains and the sites of potential amino acid substitutions caused by non-synonymous coding SNPs. Non-synonymous coding SNPs within *App* were obtained from the Center for Genome Dynamics (http://cgd.jax.org/cgdsnpdb). The likelihood scores for these SNPs to cause deleterious effects within the associated protein's structure using PROVEAN and the PANTHER Classification System are displayed. Using PROVEAN, a score of ≤−2.5 indicates a functional effect on the protein. For the PANTHER algorithm, a subSPEC (substitution position-specific evolutionary conservation) score of −3 corresponds to a 50% probability that a score is deleterious (Pdeleterious = 0.5). Likely deleterious values have been bolded.

As shown in Figure 4, the haplotype structure of *App* illustrates similar groupings of sensitive and resistant strains as the one seen for the top peak (Figure 4A). Additionally, the gene does contain non-synonymous coding SNPs (Figure 4B), which introduce the following amino acid sequence changes in the protein: D516E, A480V, D309E, and G221S. Using PROVEAN and PANTHER algorithms, A480V and D309E were classified as likely deleterious to App (Figure 4C). Finally, based on literature review, although associated mutations and differences in expression in *App* have been historically linked primarily to Alzheimer's disease, there is evidence of a role for *App* in cytotoxicity involving chemotherapeutics (Uberti et al., 2007; Woods and Padmanabhan, 2013).

**Figure 5:**
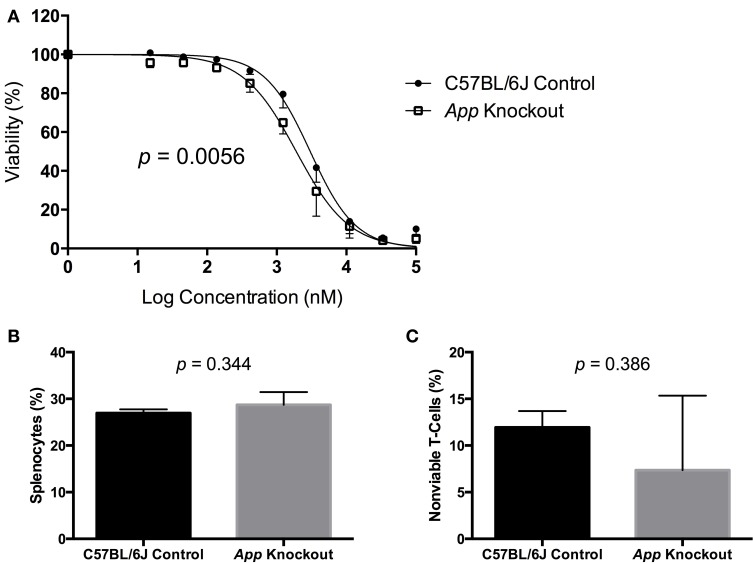
***In vitro* validation of *App* in T-cell toxicity following idarubicin exposure**. Dose-response curves **(A)** and baseline splenic T-cell composition **(B)** and non-viable T-cells **(C)** are shown. Dose-response curves were generated following exposure of splenic T-cells from C57BL/6J control mice (*N* = 3) and *App* knockout mice (*N* = 3) to idarubicin. A significant shift to the left was observed in *App* knockout cells as calculated using a partial *F*-test (*p* = 0.0056). At the zero dose, the relative splenic T-cell composition and viability of *App* knockout vs. control mice were not statistically different using a *t*-test (*p* > 0.05, respectively *p* = 0.344 and *p* = 0.386).

A gene validation study was performed using splenocytes from *App* knockout (B6.129S7-Apptm1Dbo/J) and C57BL/6J control mice, subjected to the same conditions within our cellular screen. Without drug exposure, the relative splenic T-cell composition and viability of *App* knockout vs. control mice were not statistically different using a *t*-test (*p* > 0.05, respectively *p* = 0.344 and *p* = 0.386). However, the mean viability of T-cells from *App* knockout mice was more than that of the control mice following exposure to idarubicin (Figure 5). Given that the log_10_(IC_50_) value for the control mice (3.48, 95% CI: 3.401–3.56) is significantly higher than the log_10_(IC_50_) value for the *App* knockout mice (3.28, 95% CI: 3.18–3.38), this result suggests that susceptibility to idarubicin-induced cytotoxicity on T-cells is greater with the absence of App (*p* < 0.05). Additionally, the knockout of *App* was significantly associated with increased cell toxicity as observed by the shift to the left in the dose-response curve from the control mice (partial *F*-test, *p* = 0.0056, Figure 5).

## Discussion

This investigation aimed to uncover genetic components of the normal immune system's sensitivity to chemotherapeutic agents. The importance of this comes from studies that implicate the uncompromised immune system in the efficacy of chemotherapeutic treatments (Hanahan and Weinberg, 2011). In this study, we assessed the resilience of immune function cells to potentially toxic drugs, including anticancer agents. As this investigation is difficult to conduct in human patients, we proposed a model system to examine cytotoxicity in healthy immune cells from inbred strains of mice with the objective of identifying genetic biomarkers of immune cytotoxicity (Frick et al., 2015).

Recently, standardization of pharmacogenomic screening has come under intense scrutiny, necessitating improvements in the design, application, and implementation of robust assays for phenotypic measurement (Haibe-Kains et al., 2014; Hatzis et al., 2014). For these GWAS, we examined IC_50_ values, AUC values, and individual viability concentrations (only results from the GWAS for individual viability concentrations are shown here). It is often not clear how to best represent the phenotype data when multiple varying dose-response curves are involved, and all three measurements used for GWAS present their own challenges and benefits. In this study, IC_50_ could not be estimated in some cases as necessary concentrations for 50% viability were outside of our selected, generic concentration range (15 nM–100 μM) and were far beyond physiological boundaries. While IC_50_ is a biologically relevant measurement if slopes are comparable, it can be regarded as a moving target and differs based on software and equations used to fit the dose-response curve. AUC measures can always be estimated from the dose-response curve and all points are used in data analysis. However, the appropriateness of this model in regards to its biology has been questioned (Brown et al., 2011; Fallahi-Sichani et al., 2013). The viability concentrations, which were located on either side of the mean logarithmic IC_50_ for all strains, provided precise, replicable, and robust measurements and the necessary interstrain variation for GWAS compared to IC_50_ and AUC values. Therefore, the cell viability data from these viability concentrations were used to compute the genome-wide significant QTL.

In this study, we uncovered four genome-wide significant QTL that were identified with two different GWA algorithms (EMMA and SNPster) using the same genotype and phenotype data. Because the two mapping approaches determine QTL using different methodologies, the use of both algorithms potentially helps to minimize identification of false positive QTLs. Results from both analyses identified four loci containing 25 candidate genes. These candidate genes subsequently underwent careful inspection to examine as much available data that can be garnered to rank these genes and select a “most likely” candidate for validation.

Here, we selected one gene for a validation study; *App* was validated *ex vivo* using knockout and control mice. *App* knockout mice are commercially available and viable, and only with a concurrent knockout of *Aplp2* (amyloid precursor-like protein 2) is this loss of function perinatally lethal. The downstream processing of App is fairly complex, and the role of domains in addition to the plaque forming β-App, typically associated with Alzheimer's disease, is still under investigation. *App* consists of multiple domains (i.e., A4, N-terminal heparin-binding, copper-binding, KU, E2, β-App, and amyloid C-terminal domains as shown in Figure 4B) with numerous cleavage sites (Sherry et al., 2001). Alzheimer's disease has been suggested to result from an imbalance in the production and clearance of β-APP However, additional theories have been proposed, for instance suggesting β-APP is a marker of oxidation rather than a symptom of neurodegeneration (Dong et al., 2012).

*APP* has been studied primarily in the context of Alzheimer's disease, but knowledge of other biological functions has not been as well studied. This gene is ubiquitously expressed, suggesting roles outside of various neuronal functions and potentially in diseases likely to occur due to aberrant processes that are typically associated with *APP*. The extracellular portion of the protein has been implicated in cell adhesion, signaling, and growth, and the intracellular portion has been associated with cell signaling and apoptosis (Thinakaran and Koo, 2008; Dawkins and Small, 2014). Overexpression of *APP*, particularly the soluble N-terminal ectodomain (sAPP), has been linked to carcinogenesis, including cancers originating from the nasopharynx, oral cavity, lung, breast, thyroid, parathyroid, colon, testicles, and pancreas (Takagi et al., 2013; Yamada et al., 2013). Ryan et al. exposed rat hippocampus slice cultures to sAPP, which elicited an inflammatory and immune gene response that was suggested to cause a neuroprotective environment. Apoptotic pathways were downregulated, while cell proliferation and survival pathways were upregulated (Ryan et al., 2013). In addition, *APP* expression is indirectly linked to Ras/MAPK and PI3K/Akt pathways, which are often upregulated in various cancers (Ruiz-León and Pascual, 2004). These findings suggest a potential role of *APP* in cellular processes involved in cancer or in chemotherapy response.

Epidemiologically, an inverse comorbidity with cancer was found in two studies of 500 patients with Alzheimer's disease (Tabarés-Seisdedos and Rubenstein, 2013). This inverse correlation was hypothesized to be driven by molecular processes common to CNS disorders and cancer. Ibáñez et al. found a significant overlap between genes (e.g., *PIN1, Wnt* pathway, p53 pathway, and pathways related to protein folding and folding degradation) upregulated in CNS disorders (i.e., Alzheimer's disease, Parkinson's disease, and schizophrenia) and genes downregulated in cancer (i.e., lung, prostate, and colorectal cancers) and vice versa (Ibáñez et al., 2014). *APP* also may be affected by anticancer chemotherapeutics. A recent clinical observational study indicated that the risk of Alzheimer's disease was reduced following the administration of anticancer chemotherapy (Stong, 2013).

Additionally, in CHO cells, carmustine administration reduced β-APP and was suggested to cause altered intracellular trafficking and processing of APP with an increase in sAPP and immature APP levels at the cell surface (Hayes et al., 2013). *APP* overexpressing cell lines have also been found to have a higher resistance to cytotoxicity; overexpression of wild-type *APP* in HEK cells resulted in a conformational change in p53 and a subsequent reduced sensitivity to doxorubicin (Uberti et al., 2007). The link between APP and p53 has been previously suggested, and APP has been proposed to activate gene transcription in a similar way as Notch, a protein with roles in cell differentiation, cell proliferation, neuronal function, and T-cell lineage commitment (Thinakaran and Koo, 2008). Altogether, these findings suggest a potential role of APP in cancer and in anticancer drug response.

We have not yet determined the mechanism as to why a lack of *App* leads to enhanced toxicity to idarubicin. From the findings discussed above, we can hypothesize that an increase in wild type APP and subsequent sAPP leads to a decrease in functional proteins within the p53 pathway, which causes a downregulation in apoptotic processes, upregulation in cell survival, and subsequent resistance to toxic insults such as treatment with anticancer drugs. This situation could present a clinical conundrum in how to treat patients with cancers overexpressing *APP* and thus warrants further investigation (Lanni et al., 2012).

Genomic differences in molecular machinery processing APP could add additional layers of complexity. Full-length APP is processed by α-, β-, and γ-secretases to yield β-APP or sAPP in the extracellular domain and complementary membrane-tethered fragments that are further cleaved into smaller peptides. Alternative splicing generates several APP isoforms, often tissue-specific, ranging from 365 to 770 amino acids. The exact nature of these fragments is difficult to determine, but they can have functions independent of the parent protein. The effect of idarubicin on the profile of alternative APP variants and APP cleavage products remains to be determined. A number of post-translational modifications also occur in APP; APP is extensively glycosylated in extra- and intracellular domains and is phosphorylated at several residues in its cytoplasmic domain, which interacts with multiple proteins (Muresan and Ladescu Muresan, 2015). The intracellular concentrations of active drugs may be affected by these modifications in general (Marin et al., 2014); however, the effect of drugs, particularly idarubicin, on these processes in APP warrants further investigation.

In this study, the expression of *App* in the spleen and other immune cells did not correlate with the interstrain sensitivity of T-cells to idarubicin. Thus, the effect of *App* on anticancer cytotoxicity is not likely driven by *App* expression in this study. An additional study to explore the mechanism of *App*'s effect on idarubicin toxicity may include creating specific polymorphisms introducing the potentially deleterious non-synonymous coding changes mentioned previously to see how the viability of T-cells exposed to idarubicin is affected. Furthermore, we have additional candidate genes from our screen for future validation. Of particular interest, *Ppfia1* and *Ppfibp1* were found using the viability of B-cells exposed to doxorubicin and idarubicin, respectively. These genes encode liprin-alpha-1 and liprin-beta-1, members of the LAR protein tyrosine phosphatase-interacting protein family, which orchestrate cell-matrix interactions (http://omim.org/, 2014). Future studies examining the roles of *Ppfia1* and *Ppfibp1* in immune-mediated cytotoxicity are needed to better understand this finding.

We achieved our aims by demonstrating that we can identify genes implicated in the immune cell survival after treatment with chemotherapeutic agents. Using a cellular screening approach, we identified and subsequently validated a gene involved in cytotoxic T-cell sensitivity to idarubicin. Further work would be required to define the precise mechanism by which *APP* mediates this sensitivity. In addition, it is very unlikely that this is the only gene that impacts the variable response to this chemotherapeutic agent. We identified additional candidate genes of interest that would also require a validation process, with the ultimate goal of translating these findings to clinical practice.

### Conflict of interest statement

Dr. Kristy L. Richards received an honorarium from Celgene and is on the advisory board for Genentech. The authors declare that the research was conducted in the absence of any commercial or financial relationships that could be construed as a potential conflict of interest.

## Supplementary material

Supplementary Table 1 **Candidate genes from the phenotypic screen**. The gene symbol, full name, and genome-wide significant -log (*p*) score at the QTL peak are represented. The table also indicates if the gene is expressed (>50) or differentially expressed (>2 fold difference in expression levels) and if expression correlates with its phenotype (*p* < 0.05) within the spleen, CD4+ T-cells, CD4+ Th1 T-cells, and macrophages. Furthermore, the presence of a haplotype match with the QTL, potentially deleterious non-synonymous coding SNPs, involvement in biological pathways, and association with the immune system or anthracyclines is specified for each candidate gene.

Supplementary Figure 1 **Schematic for candidate gene prioritization**.
